# Supplementary material for: Charting the Ethical Frontier in Newborn Screening Research: Insights from the NBSTRN ELSI Researcher Needs Survey
Source: Int J Neonatal Screen. 2024 Sep 19;10(3):64. doi: 10.3390/ijns10030064 (PMC11417897; doi:10.3390/ijns10030064)
Supplement: Supplementary file 1 [file IJNS-10-00064-s001.zip › IJNS-2946692-supplementary.pdf]

Participant ID	1. Which best describes your interest and affiliation with NBSTRN?	2. Which area best describes your role in newborn screening research?	3. Please select the affiliation that best aligns with your newborn screening research:	4. How many years have you been involved in newborn screening research?	5. When encountering legal or ethical issues related to newborn screening research, where do you typically seek information or guidance? Please select all that apply:	5. If other, please specify.	6. Are you familiar with the ethical and legal resources provided by NBSTRN?	7. Given the definition of privacy as 'preventing others from knowing information that you do not wish for them to know', on a scale of 1 to 5, how important of an ethical issue	8. Using the above definition of privacy, how important is it that the NBSTRN address privacy in big data health research by developing new (or emphasize existing) policies and/or best	9. How important is it that NBSTRN address informed consent for newborn screening research by developing new (or emphasize existing) policies and/or best practices?	10. How interested would you be in training or educational activities related to ethical issues in newborn screening research?	11. Please rank your interest in the following activities for learning about ethical issues in newborn screening research, where your first choice indicates the activity you are most interested in,	11. Please rank your interest in the following activities for learning about ethical issues in newborn screening research, where your first choice indicates the activity you are most interested in,	11. Please rank your interest in the following activities for learning about ethical issues in newborn screening research, where your first choice indicates the activity you are most interested in,	11. Please rank your interest in the following activities for learning about ethical issues in newborn screening research, where your first choice indicates the activity you are most interested in,	11. Please rank your interest in the following activities for learning about ethical issues in newborn screening research, where your first choice indicates the activity you are most interested in,	11. Please rank your interest in the following activities for learning about ethical issues in newborn screening research, where your first choice indicates the activity you are most interested in,	12. What are the ethical, legal, or social issues that you face in your own work? Explain.	13. How interested would you be in a research ethics consultation service that helps answer ethical issues around the ethical issues surrounding NBS research?
1	Other	Staff	Nonprofit organization research	3 - 5 years	Institutional lawyers or ethics committees within your organization		Yes, I am aware of the NBSTRN ethical/legal resources	5	5	5	Very interested	4th	3rd	1st	2nd	6th	5th	Not sure	very interested
2	State NBS Program	Clinical Care	Clinical research	3 - 5 years	State lawyers specializing in healthcare law		No, I am not aware of the NBSTRN ethical/legal resources.	4	3	4	Very interested	4th			3rd	2nd	1st		Somewhat interested
3	State NBS Program	State Program	NBS state program research	1 - 3 years			Yes, I am aware of the NBSTRN ethical/legal resources	5	4	3									
4	Other	Other	Pharmaceutical industry (pharma)	Less than 1 year	Other		No, I am not aware of the NBSTRN ethical/legal resources.	3	5	5	Somewhat interested	1st	2nd	4th	3rd	6th	5th		Somewhat interested
5	State NBS Program	State Program	NBS state program research	More than 10 years	Institutional lawyers or ethics committees within your organization		Yes, I am aware of the NBSTRN ethical/legal resources	5	5	5	Very interested	4th	1st	2nd	3rd			informed consent	Somewhat interested
6	State NBS Program	Staff	NBS state program research	More than 10 years	State lawyers specializing in healthcare law		No, I am not aware of the NBSTRN ethical/legal resources.	5	5	5	Very interested	1st	2nd	3rd	4th			Screening for new genetic diseases	Somewhat interested
7	Advocacy Group	Advocacy Group	NBS state program research	5 - 10 years	Other	I do both bio and legal research myself and include research to primary sources and contacts with individuals with shown skills in the areas in question.	Yes, I am aware of the NBSTRN ethical/legal resources	4	4	4	Very interested	2nd	3rd	4th	1st			Focus is the application of whole genome sequencing (WGS) in primary health care offices, and a particular focus of using WGS as a supplement to newborn screening process.	Somewhat interested
8	Clinician	Researcher	Translational research	3 - 5 years	Other		No, I am not aware of the NBSTRN ethical/legal resources.	5	3	4	Somewhat interested	2nd	1st	3rd	4th	6th	5th		Somewhat interested
9	Clinician	Researcher	Academic/university research	More than 10 years	Institutional lawyers or ethics committees within your organization		No, I am not aware of the NBSTRN ethical/legal resources.	5	5	4	Somewhat interested	3rd	1st	2nd	4th	6th	5th	large scale interventions that make individual consent difficult.	very interested
10	State NBS Program	State Program	NBS state program research	More than 10 years	State lawyers specializing in healthcare law		Yes, I am aware of the NBSTRN ethical/legal resources	2	3	4	Not at all interested	2nd	1st	4th	3rd	5th		Various ELSI issues re: storage and use of residual DBS	Not at all interested
11	State NBS Program	State Program	NBS state program research	More than 10 years	Institutional lawyers or ethics committees within your organization		No, I am not aware of the NBSTRN ethical/legal resources.	4	3	4	Somewhat interested	3rd	1st	4th				Unable to convince State Public Health officials that providing deidentified case level data is OK. Also ran into problems where we wanted to use known trait data to see if traits were being recorded in child's medical record. Got consent from hospital system but not state.	Somewhat interested
12	Researcher	Researcher	Academic/university research	1 - 3 years	Other	USPSTF	No, I am not aware of the NBSTRN ethical/legal resources.	2	2	3	Very interested	1st	3rd	2nd	4th			I also work on ELSI of adult screening and gene editing of newborn screening conditions. I am also interested in ethical justifications of waived consent for public health surveillance/research.	Somewhat interested
13	Clinician	Clinical Care	Academic/university research	5 - 10 years	Institutional lawyers or ethics committees within your organization		No, I am not aware of the NBSTRN ethical/legal resources.	5	5	5	Very interested	4th	3rd	2nd	1st				very interested
14	State NBS Program	State Program	NBS state program research	5 - 10 years	Institutional lawyers or ethics committees within your organization		No, I am not aware of the NBSTRN ethical/legal resources.	5	5	4	Very interested	3rd	1st	2nd	4th	6th	5th	Parent concerns about individual level privacy	Not at all interested
15	Researcher	Researcher	Academic/university research	3 - 5 years	Nonprofit organizations such as APHL		No, I am not aware of the NBSTRN ethical/legal resources.	5	5	4	Somewhat interested	1st	3rd		2nd		6th	I do not work directly with patients. While I have opinions and participate in discussions relating to these policies, my work will not directly impact newborn screening ELSI related issues.	Not at all interested
16	Researcher	Researcher	Academic/university research	3 - 5 years	Institutional lawyers or ethics committees within your organization		No, I am not aware of the NBSTRN ethical/legal resources.	4	4	4	Somewhat interested	1st	2nd	3rd	4th		6th		very interested
17	State NBS Program	State Program	NBS state program research	3 - 5 years	Institutional lawyers or ethics committees within your organization		No, I am not aware of the NBSTRN ethical/legal resources.	5	5	5	Very interested	3rd	1st	2nd	4th			Research on marginalized populations - working with patient advocacy groups, etc.	Somewhat interested
18	State NBS Program	Clinical Care	NBS state program research	3 - 5 years	Colleagues doing legal ethical research		Yes, I am aware of the NBSTRN ethical/legal resources	4	4	5	Somewhat interested	1st							

19	Clinician	Clinical Care	Academic/university research	5 - 10 years	Colleagues doing legal ethical research		No, I am not aware of the NBSTRN ethical/legal resources.	4	4	3	Somewhat interested	1st							Somewhat interested
20	State NBS Program	State Program	NBS state program research	More than 10 years	Institutional lawyers or ethics committees within your organization		No, I am not aware of the NBSTRN ethical/legal resources.	4	5	5	Somewhat interested	1st	3rd	2nd	4th			Lack of ability to do any research as State has code preventing "any research" on Newborn Screening without consent being obtained. The state often doesn't understand what research is and simple straight forward chart reviews to see if trait status is marked/newborn screening results in EMR is prohibited. On a personal issue the more Newborn Screening incorporated sequencing into it's standard practice I believe true INFORMED consent should be required - I know more may refuse but the amount of data we are accumulating/will accumulate should require consent.	Somewhat interested
21	Other	Clinical Care	Clinical research	1 - 3 years	Institutional lawyers or ethics committees within your organization		No, I am not aware of the NBSTRN ethical/legal resources.	3	3	4	Very interested	2nd	3rd	1st	4th				Not at all interested
22	State NBS Program	Staff		More than 10 years	Colleagues doing legal ethical research		No, I am not aware of the NBSTRN ethical/legal resources.	5	5	3	Somewhat interested	1st	3rd	2nd	4th	5th	6th		Not at all interested
23	Advocacy Group	Advocacy Group	Nonprofit organization research	More than 10 years	Colleagues doing legal ethical research		No, I am not aware of the NBSTRN ethical/legal resources.	4	5	5	Very interested	1st	2nd	3rd	4th				Not at all interested
24	Other	Researcher	Academic/university research	5 - 10 years	Institutional lawyers or ethics committees within your organization		Yes, I am aware of the NBSTRN ethical/legal resources	5	5	4	Very interested	3rd	1st	2nd	4th	6th	5th	Consent, use of residual DBS	Somewhat interested
25	State NBS Program	State Program	NBS state program research	3 - 5 years	Nonprofit organizations such as APHL		Yes, I am aware of the NBSTRN ethical/legal resources	5	5	3	Somewhat interested	1st	2nd	3rd	6th				Not at all interested
26	Patient	Parent	Other	More than 10 years	Other		Yes, I am aware of the NBSTRN ethical/legal resources	4	5	5	Somewhat interested	4th	1st	3rd	2nd				Somewhat interested
27	Researcher	Researcher	Translational research	5 - 10 years	Institutional lawyers or ethics committees within your organization		No, I am not aware of the NBSTRN ethical/legal resources.	4	5	5	Somewhat interested	1st	2nd	4th	3rd	6th	5th		Somewhat interested
28	Researcher	Researcher	Academic/university research	1 - 3 years	Colleagues doing legal ethical research		No, I am not aware of the NBSTRN ethical/legal resources.	5	5	4	Somewhat interested	1st	2nd	3rd	4th	5th	6th		Not at all interested
29	Researcher	Researcher	Academic/university research	More than 10 years	Colleagues doing legal ethical research		No, I am not aware of the NBSTRN ethical/legal resources.	5	4	4	Somewhat interested	1st	3rd	2nd	4th	5th	6th	Collecting and analyzing f/u and outcome data to learn how programs can be improved.	Not at all interested
30	Researcher	Researcher	Academic/university research	More than 10 years	Institutional lawyers or ethics committees within your organization		No, I am not aware of the NBSTRN ethical/legal resources.	5	4	4	Somewhat interested	4th	2nd	1st	3rd			Informed consent in longitudinal studies that follow patients from pediatric stage through adulthood and even into advanced age. Best ways to contact once patient is adult and leaves parents (if adult contact info is missing especially), or changes contact info due to other life change, holding onto consent forms across decades (and as technology and data collection methods change) for active longitudinal studies and closed studies that may still have valuable and accessible data. Also concerns in cases where data recovery is needed if a data file is "lost" or corrupts. And ethical recruitment methods via Social Media.	very interested
31	State NBS Program	Clinical Care	Clinical research	3 - 5 years	Nonprofit organizations such as APHL		No, I am not aware of the NBSTRN ethical/legal resources.	4	4	2	Somewhat interested	4th	3rd	2nd	1st				Somewhat interested
32	Researcher	Researcher	Academic/university research	3 - 5 years	Colleagues doing legal ethical research		Yes, I am aware of the NBSTRN ethical/legal resources	4	5	5	Somewhat interested		3rd	4th	2nd				Not at all interested
33	State NBS Program	State Program	NBS state program research	Less than 1 year	Institutional lawyers or ethics committees within your organization		Yes, I am aware of the NBSTRN ethical/legal resources	5	5	5	Very interested	4th	1st	2nd	3rd	6th	5th	N/A for now	Somewhat interested

34	Researcher	Researcher	Nonprofit organization research	1 - 3 years	Colleagues doing legal ethical research		No, I am not aware of the NBSTRN ethical/legal resources.	4	5	5	Very interested	2nd	1st	3rd	5th	6th			Not at all interested
35	Researcher	Researcher	Federally funded research	3 - 5 years	Nonprofit organizations such as APHL		Yes, I am aware of the NBSTRN ethical/legal resources	4	5	5	Very interested	2nd	3rd	1st	4th	6th	5th	If we obtain cCMV positive results with an unidentified DBS specimen, how we can proceed? If the results are not reported, that newborn will suffer.	very interested
36	Advocacy Group	Advocacy Group	Clinical research	More than 10 years	Nonprofit organizations such as APHL		Yes, I am aware of the NBSTRN ethical/legal resources	4	4	4	Very interested	1st	2nd	4th	3rd				Somewhat interested
37	Clinician	Other	Other	3 - 5 years	Other	Professional societies, many sources	Yes, I am aware of the NBSTRN ethical/legal resources	4	4	4	Very interested	2nd	1st	4th	3rd		5th	Complexity of consent Downstream implications of the information, outside of medical/clinical use	Somewhat interested
38	Researcher	Researcher	Federally funded research	More than 10 years	Colleagues doing legal ethical research		No, I am not aware of the NBSTRN ethical/legal resources.	4	5	4	Somewhat interested	1st	2nd	3rd	4th				Not at all interested
39	State NBS Program	Advocacy Group	NBS state program research	1 - 3 years	Nonprofit organizations such as APHL		Yes, I am aware of the NBSTRN ethical/legal resources	5	5	4	Very interested	1st	5th	2nd	4th		6th		very interested
40	Clinician	Clinical Care	Clinical research	Less than 1 year	Institutional lawyers or ethics committees within your organization		No, I am not aware of the NBSTRN ethical/legal resources.	5	5	5	Somewhat interested	3rd	2nd	1st	4th	5th	6th		Not at all interested
41	Researcher	Researcher	Academic/university research	More than 10 years			Yes, I am aware of the NBSTRN ethical/legal resources	5	5	5	Very interested	2nd	1st	4th	3rd			The constant need to remind my co-investigators that data deposited in the LPDR is de-identfied.	very interested
42	Other	Other	Other	More than 10 years	Other	Literature based evidence.	Yes, I am aware of the NBSTRN ethical/legal resources	5	5	5	Very interested	1st	2nd	3rd	4th				very interested
43	Clinician	Researcher	Clinical research	5 - 10 years	Institutional lawyers or ethics committees within your organization		No, I am not aware of the NBSTRN ethical/legal resources.	4	4	4	Somewhat interested	1st	3rd	2nd	4th	6th	5th		Not at all interested
44	Other	Other	Nonprofit organization research	More than 10 years	State lawyers specializing in healthcare law		No, I am not aware of the NBSTRN ethical/legal resources.	3	3	3	Somewhat interested	4th	2nd	3rd	1st				Not at all interested
45	Researcher	Researcher	Federally funded research	5 - 10 years	Other	colleagues in genetics	No, I am not aware of the NBSTRN ethical/legal resources.	5	5	3	Somewhat interested	1st	2nd	3rd	4th			population pilot studies where there are more positives in a given ethnic group false positives and the evaluations needed on a population basis	very interested
46	Researcher	Researcher	Academic/university research	Less than 1 year	Colleagues doing legal ethical research		Yes, I am aware of the NBSTRN ethical/legal resources	5	5	5	Very interested	3rd	4th	1st	2nd	5th	6th		very interested
47	Researcher	Researcher	Academic/university research	Less than 1 year	Other	Legal docs	No, I am not aware of the NBSTRN ethical/legal resources.	5	5	5	Very interested	1st	4th	2nd	3rd			we use a newborn screening program that is obsolete, and not standarized. We need to actualize in nbs alternatives, and normalize the ethical and legal issues.	very interested
48	Researcher	Researcher	Nonprofit organization research	3 - 5 years	Colleagues doing legal ethical research		No, I am not aware of the NBSTRN ethical/legal resources.	5	3	4	Somewhat interested	1st	2nd	3rd	6th				Somewhat interested
49	State NBS Program	State Program	NBS state program research	5 - 10 years	Other	We have a Newborn Screening consultant we contact for issues, and we also do reach out to our legal team.	Yes, I am aware of the NBSTRN ethical/legal resources	5	5	5	Very interested	1st	2nd	3rd	4th	6th	5th	We are watching other states lawsuits regarding research, specimen storage, and data storage. We are very mindful of what might be considered unethical in research/new condition implementation practices.	very interested
50	State NBS Program	State Program	NBS state program research	Less than 1 year	State lawyers specializing in healthcare law		Yes, I am aware of the NBSTRN ethical/legal resources	5	5	5	Very interested	4th	3rd	1st	2nd	6th			Somewhat interested
51	Advocacy Group	Advocacy Group	Other	More than 10 years	Colleagues doing legal ethical research		Yes, I am aware of the NBSTRN ethical/legal resources	5	5	5	Somewhat interested	1st	2nd	5th	3rd				Somewhat interested
52	Clinician	Other	Academic/university research	More than 10 years	Institutional lawyers or ethics committees within your organization		Yes, I am aware of the NBSTRN ethical/legal resources	4	4	4	Very interested	2nd	1st	3rd	4th				Not at all interested

53	Advocacy Group	Advocacy Group	Other	3 - 5 years	Nonprofit organizations such as APHL		Yes, I am aware of the NBSTRN ethical/legal resources	5	5	5	Very interested	1st	5th	4th	2nd		3rd	getting past hold power in US Senate	Somewhat interested
54	State NBS Program	Advocacy Group	Nonprofit organization research	3 - 5 years	Institutional lawyers or ethics committees within your organization		No, I am not aware of the NBSTRN ethical/legal resources.	3	2	4	Somewhat interested		2nd	1st	3rd	5th	4th	Advising state program on policy recommendations, advocating for changing processes around parental refusal to make them more burdensome on parents and adding more friction into the refusal option	very interested
55	Researcher	Researcher	Academic/university research	More than 10 years	Other	All of the above. Note: Select all that apply doesn't work.	Yes, I am aware of the NBSTRN ethical/legal resources	5	5	5	Somewhat interested	3rd	1st		2nd				Not at all interested
56	Researcher	Researcher	Clinical research	1 - 3 years	Colleagues doing legal ethical research		No, I am not aware of the NBSTRN ethical/legal resources.	3	3	3	Somewhat interested	1st	6th					Marginalization of those in minority groups	Somewhat interested
57	Clinician	Researcher	Nonprofit organization research	More than 10 years	Other	all but APHL	Yes, I am aware of the NBSTRN ethical/legal resources	3	5	5	Somewhat interested	1st	6th	5th	4th			informed consent for research	Not at all interested
58	Clinician	Clinical Care	Academic/university research	More than 10 years	Colleagues doing legal ethical research		No, I am not aware of the NBSTRN ethical/legal resources.	5	4	4	Very interested		4th	5th	3rd			.	Somewhat interested
59	Researcher	Researcher	Federally funded research	Less than 1 year	Colleagues doing legal ethical research		No, I am not aware of the NBSTRN ethical/legal resources.	5	5	4	Very interested	3rd	1st	2nd	4th	5th	6th		very interested
60	State NBS Program	State Program	NBS state program research	3 - 5 years	Institutional lawyers or ethics committees within your organization		No, I am not aware of the NBSTRN ethical/legal resources.	5	1	1	Not at all interested	3rd	4th	5th	6th	1st	2nd	IVDR regulation	Not at all interested
61	Clinician	Clinical Care	Academic/university research	More than 10 years	Other	all above, the system can only tick single answer	Yes, I am aware of the NBSTRN ethical/legal resources	4	4	4	Very interested	2nd	1st	4th	3rd				very interested
62	State NBS Program	State Program	NBS state program research	1 - 3 years	Colleagues doing legal ethical research		No, I am not aware of the NBSTRN ethical/legal resources.	4	4	4	Somewhat interested	1st	2nd	4th	3rd	5th	6th	a lot of agreements and consents needed on privacy, data sharing and other things before you can use stored DBS for research projects.	Somewhat interested
63	State NBS Program	State Program	NBS state program research	More than 10 years	State lawyers specializing in healthcare law		No, I am not aware of the NBSTRN ethical/legal resources.	5	5	5	Somewhat interested	3rd	4th	1st	2nd	6th	5th	equitable care in NBS, both is what babies get screened and in delivery of follow-up care	Not at all interested
64	State NBS Program	State Program	NBS state program research	3 - 5 years	Institutional lawyers or ethics committees within your organization		No, I am not aware of the NBSTRN ethical/legal resources.	5	5	5	Somewhat interested	1st						Mandatory screen with no opt out	Somewhat interested
65	State NBS Program	State Program	NBS state program research	More than 10 years	Institutional lawyers or ethics committees within your organization		Yes, I am aware of the NBSTRN ethical/legal resources	5	5	4	Very interested	4th	3rd	2nd	1st				Not at all interested
66	Researcher	Clinical Care	Academic/university research	More than 10 years	Other	The literature for starters, then the university IRB, then major professional groups, SIMD, APHL, ACMG	No, I am not aware of the NBSTRN ethical/legal resources.	5	4	5	Very interested	1st	2nd	5th	4th			Designing studies that adequately protect the privacy of patients, families and providers.	Not at all interested
67	State NBS Program	Clinical Care	NBS state program research	5 - 10 years	Institutional lawyers or ethics committees within your organization		No, I am not aware of the NBSTRN ethical/legal resources.	4	5	5	Very interested	4th	2nd	1st	3rd	5th			Somewhat interested
68	Parent	Advocacy Group	Nonprofit organization research	5 - 10 years	Nonprofit organizations such as APHL		Yes, I am aware of the NBSTRN ethical/legal resources	4	4	4	Very interested	2nd	1st	3rd	4th			Expansion of NBS for leukodystrophies that may have later onset	Somewhat interested
69	Parent	State Program	Nonprofit organization research	3 - 5 years	Institutional lawyers or ethics committees within your organization		No, I am not aware of the NBSTRN ethical/legal resources.	2	4	3	Somewhat interested	1st	2nd	4th	3rd				Somewhat interested
70	State NBS Program	State Program	NBS state program research	5 - 10 years	Institutional lawyers or ethics committees within your organization		No, I am not aware of the NBSTRN ethical/legal resources.	4	4	5	Very interested	4th	1st	2nd	3rd			We perform testing for multiple states and each state has its own system for reviewing and approving research requests. Understanding these systems is critical to ensure that all research is approved in advance of any sharing of data or blood spots.	Somewhat interested
71	Researcher	Researcher	Academic/university research	More than 10 years	Institutional lawyers or ethics committees within your organization		No, I am not aware of the NBSTRN ethical/legal resources.	5	5	5	Very interested	1st	2nd	3rd	4th	5th	6th		Somewhat interested

72	Researcher	Researcher	Pharmaceutical industry (pharma)	More than 10 years	Colleagues doing legal ethical research		Yes, I am aware of the NBSTRN ethical/legal resources	5	3	3	Very interested	1st	2nd	3rd	4th			Having potentially actionable genetic information (including potentially life saving interventions) that cannot be reported because of privacy issues, insufficient informed consent or genetic testing laboratory contracts. Also diagnosing patients and then having nothing to offer when patients do not meet clinical trial inclusion criteria and there is no option for expanded access. Also placebo groups for clinical trials for terminal conditions.	Somewhat interested
73	Researcher	Researcher	Translational research	1 - 3 years	Colleagues doing legal ethical research		No, I am not aware of the NBSTRN ethical/legal resources.	4	4	5	Very interested	2nd	1st	4th	3rd	6th	5th	Researching issues like information needs, informed consent, data sharing for research	Somewhat interested
74	Other	Other	Technology development research	5 - 10 years	Institutional lawyers or ethics committees within your organization		No, I am not aware of the NBSTRN ethical/legal resources.	5	5	5	Somewhat interested	2nd	1st	3rd	4th	5th	6th	Possibility to identify origin of DNA samples using SNP is a problem when running research.	Somewhat interested
75	Researcher	Researcher	Academic/university research	More than 10 years	Colleagues doing legal ethical research		No, I am not aware of the NBSTRN ethical/legal resources.	2	2	1	Not at all interested	1st	2nd	3rd	4th			I am semi-retired. I find that IRBs are a time-consuming and morale-sapping activity. Too much attention is paid to form and to issues at the asymptote of concern.	Not at all interested
76	Clinician	Clinical Care	Academic/university research	3 - 5 years	Institutional lawyers or ethics committees within your organization		No, I am not aware of the NBSTRN ethical/legal resources.	3	3	2	Somewhat interested	3rd	1st	2nd	4th				Somewhat interested
77	State NBS Program	Researcher	NBS state program research	More than 10 years	Colleagues doing legal ethical research		No, I am not aware of the NBSTRN ethical/legal resources.	4	5	4	Very interested	1st	4th	3rd	5th	6th	2nd	Whether or not genetic results should be available to heterozygote carrier infants and/or their parents.	Somewhat interested
78	State NBS Program	State Program	NBS state program research	5 - 10 years	Other	The top 3- cannot select more than one option for this question.	Yes, I am aware of the NBSTRN ethical/legal resources	5	5	5	Very interested	4th	3rd	1st	2nd			The current issue is of sickle cell trait results and the NCAA requirement for "proof of status". Does the NBS result qualify to meet that "proof".	Somewhat interested
79	State NBS Program	State Program	NBS state program research	More than 10 years	Institutional lawyers or ethics committees within your organization		Yes, I am aware of the NBSTRN ethical/legal resources	5	5	5	Somewhat interested	1st	3rd	2nd	4th				Somewhat interested
80	Clinician	Clinical Care	Clinical research	More than 10 years	Institutional lawyers or ethics committees within your organization		No, I am not aware of the NBSTRN ethical/legal resources.	2	4	4	Somewhat interested	1st	3rd	2nd	4th				Somewhat interested
81	Researcher	Researcher	Academic/university research	More than 10 years	Institutional lawyers or ethics committees within your organization		Yes, I am aware of the NBSTRN ethical/legal resources	5	4	5	Very interested	1st	2nd	3rd	4th			NBS research consent	Somewhat interested
82	State NBS Program	State Program	Clinical research	3 - 5 years	Colleagues doing legal ethical research		No, I am not aware of the NBSTRN ethical/legal resources.	4	4	4	Somewhat interested	1st	3rd	2nd	4th	5th	6th	In my clinical research, it is always a struggle to be able to identify families/obtain consent to be recontacted (to be consented for clinical NBS research using methods like surveys and interviews) as our NBS program is set up with an opt-out approach.	Not at all interested
83	State NBS Program	State Program	NBS state program research	More than 10 years	State lawyers specializing in healthcare law		No, I am not aware of the NBSTRN ethical/legal resources.	5	5	5	Very interested	1st	2nd	3rd					Somewhat interested
84	Researcher	Researcher	Other	3 - 5 years	Other	combo of colleagues and direct reserach. I find that there is a lot of scope creep conservatism in the field and its overly restricting, while loosing sight of the intent and letter of initial guidance.	No, I am not aware of the NBSTRN ethical/legal resources.	5	4	4	Somewhat interested	5th	3rd	4th	1st			all of the above of course depend on the perspective and rigor of the material. I rank discussion as the most important because it has the best chance of unearthing real world stuff, especially in grey areas vs taking the easy way out where everything is on one side of a very conservatively drawn line.	Somewhat interested
85	State NBS Program	State Program	NBS state program research	More than 10 years	State lawyers specializing in healthcare law		No, I am not aware of the NBSTRN ethical/legal resources.	5	4	4	Very interested	4th	3rd	2nd	1st	6th	5th	Appropriate use of residual blood specimens. Unintended consequences of not NBS technologies. Public perceptions of NBS research.	Somewhat interested
86	State NBS Program	State Program	NBS state program research	Less than 1 year	Institutional lawyers or ethics committees within your organization		No, I am not aware of the NBSTRN ethical/legal resources.	5	5	5	Very interested	1st	4th	3rd	2nd	6th	5th		Somewhat interested
87	Parent	Parent	Nonprofit organization research	More than 10 years	Nonprofit organizations such as APHL		No, I am not aware of the NBSTRN ethical/legal resources.	3	3	4	Not at all interested	4th	2nd	3rd	1st				Not at all interested
88	Researcher	Researcher	Translational research	More than 10 years	Nonprofit organizations such as APHL		Yes, I am aware of the NBSTRN ethical/legal resources	5	5	5	Somewhat interested	3rd		2nd	1st			The basic principles of ethical, legal, and equitable standards must always be observed in public health research.	Not at all interested
